# Reasons and experience for patients with amyotrophic lateral sclerosis using traditional Chinese medicine: a CARE-TCM based mixed method study

**DOI:** 10.1186/s12906-024-04513-2

**Published:** 2024-06-12

**Authors:** Qiuyang Jia, Yuebo Song, Chi Zhang, Mingxuan Li, Luda Feng, Kazuo Sugimoto, Xuebin Zhang, Jia Liu, Ying Gao

**Affiliations:** 1 Dongzhimen Hospital, Beijing University of Chinese Medicine, Beijing, China; 2 Institute for Brain Disorders, Beijing University of Chinese Medicine, Beijing, China; 3 Gansu provincial Hospital of traditional Chinese medicine, Gansu, China; 4 Dongfang Hospital, Beijing University of Chinese Medicine, Beijing, China; 5 Key Laboratory of Chinese Internal Medicine of Ministry of Education and Beijing, Dongzhimen Hospital, Beijing University of Chinese Medicine, Beijing, China; 6 Institute for Brain Disorders, Beijing University of Chinese Medicine Dongzhimen Hospital, Beijing University of Chinese Medicine, Beijing, 100700 China

**Keywords:** Traditional Chinese medicine, Herbal medicine, Amyotrophic lateral sclerosis, Mixed method study

## Abstract

**Background and aim:**

Traditional Chinese medicine (TCM) is widely used by patients with amyotrophic lateral sclerosis (ALS). However, their reasons and experience in using TCM have received insufficient attention. Therefore, we conducted a mixed method study to gain insights into this issue.

**Materials and methods:**

This study was conducted on the basis of the China Amyotrophic Lateral Sclerosis Registry of Patients with Traditional Chinese Medicine (CARE-TCM). Data were collected from Dongzhimen Hospital through a mixed method approach, including a questionnaire and a semi-structured interview. Patients with ALS who were using TCM when they were initially registered with CARE-TCM and who had been followed-up for over six months were recruited. The questionnaires’ outcomes were statistically outlined, and the interview transcripts were thematically analysed to identify themes and sub-themes.

**Results:**

Fifty-two and sixteen patients were included in the questionnaire and semi-structured interview groups, respectively. Patients used TCM with the hope of regulating their body holistically to improve nonmotor symptoms and quality of life (QOL). Those who recognised TCM as ineffective tended to discontinue it after a three-month trial period. Although quality was a major concern, herbal medicine (HM) was the most frequently used modality among all participants (*n* = 52), with the majority (*n* = 44, 84.6%) continuing to use it. Patients emphasised in-person consultations as a crucial part of TCM treatment. However, the disability caused by disease often made this interaction unattainable.

**Conclusion:**

Nonmotor symptoms and QOL hold substantial importance for patients with ALS using TCM. HM is a more suitable modality than other TCM treatment modalities, but patients are facing challenges in seeking HM treatment. It is necessary to promote the implementation of hierarchical diagnosis and treatment, thus making TCM more accessible.

**Trial registration:**

ClinicalTrials.gov identifier: NCT04885374 (registered on May 13, 2021).

**Supplementary Information:**

The online version contains supplementary material available at 10.1186/s12906-024-04513-2.

## Introduction

Amyotrophic lateral sclerosis (ALS) is a rare but fatal neurodegenerative disease characterised by progressive limb atrophy and paralysis [1]. Death often occurs within three to five years from respiratory failure due to diaphragm paralysis [2]. In China, only riluzole and edaravone have been approved by the Food and Drug Administration (FDA) for treating ALS, but their efficacy is unsatisfactory [3, 4]. Owing to limited treatment options available, patients with ALS often seek complementary therapies alongside conventional pharmaceuticals [5].

The use of Traditional Chinese medicine (TCM), including Chinese herbs, acupuncture, massage, and other modalities, is increasing worldwide, especially in China. A cross-sectional study in China indicated that over 90% of patients with ALS use TCM [6]. We established a registry study, the China Amyotrophic Lateral Sclerosis Registry of Patients with Traditional Chinese Medicine (CARE-TCM) (ClinicalTrials.gov: NCT04885374, registered on 13/05/2021) [7]. During the follow-up process, we identified that patients’ needs, perceptions, challenges, and experiences have not received adequate attention in current studies. Despite the fact that TCM has been shown to be effective in delaying ALS progression [8–10] ameliorating associated symptoms [11] in certain clinical studies [9, 11] and being supported by rodent models in terms of extending overall survival and improving brain pathology and motor function [12, 13].

Individual patient preferences considerable influence medical decision-making. Patient-centred care, evidence-based practice and value-based practices are concepts in which patient values lie at the heart of high-quality healthcare practices [14]. Nevertheless, physicians often lack awareness of patient values in TCM clinical encounters. To address this gap, we designed a questionnaire and a semi-structured interview protocol on the basis of CARE-TCM. This mixed method study aims to shed light on patients’ motivations and experiences with TCM, offering insights for future quantitative investigations. Our intention is to facilitate discussions of patient perspectives in clinical encounters and enhance physicians’ understanding of what matters most to ALS patients receiving TCM treatment.

## Methods

### Design

To reveal the reasons and experience of patients with ALS using TCM, we adopted a two-phase mixed method study. Firstly, we conducted a questionnaire regarding therapies that patients used on the basis of CARE-TCM. Secondly, a semi-structured interview was conducted following an interview outline based on the interviewers’ clinical experience. This study was conducted from December 2022 to May 2023. The interview transcripts were analysed with thematic analysis. This approach allows the investigation of phenomena in an in-depth and holistic fashion to provide insights and understanding of real-world problems, which help us better understand the thinking and behaviour of patients.

### Participants

The study was conducted at Beijing University of Chinese Medicine affiliated Dongzhimen Hospital, Beijing, China based on CARE-TCM [7]. The inclusion criteria of Phase I were as follows: (1) patients 18 years old and older; (2) patients meeting the revised El Escorial diagnostic criteria with a definite, probable, or laboratory-supported probable diagnosis of ALS [15]; (3) patients using TCM therapies when they were initially registered in CARE-TCM; and (4) patients who had been followed up in CARE-TCM for over six months. The exclusion criteria were: (1) patients with dementia or (2) uncooperative patients or caregivers during the interview. TCM therapies were defined as Chinese herbal medicine (HM), acupuncture, moxibustion, massage, and Chinese exercises like Baduanjin and Qigong. Based on the Phase I study, patients who could provide maximum information were selected to be engaged in Phase II through purposive sampling [16]. The decision on which potential participants would be most informative was made by the researchers, on the basis of what information was considered still missing to obtain the necessary data for understanding the research question. As patients with ALS often suffer from dysarthria with disease progression, the participants in this study were the patients or the caregivers accompanied by the patients. The sample size of Phase II was determined using the rule of data saturation, which is defined as two successive interviews in which no new themes are identified [17]. This study was approved by the Research Ethics Committee of Dongzhimen Hospital, Beijing University of Chinese Medicine (2021DZMEC-005-02, 2023DZMEC-100-02).

### Data collection

The participants were recruited based on the CARE-TCM. Demographic data and clinical information, including diagnosis information, treatment information, and disease progression measured by the revised amyotrophic lateral sclerosis functional rating scale (ALSFRS-R), were collected through the CARE-TCM study. In Phase I, data were gathered via an online questionnaire, whereas in Phase II, participants were expected to engage in the interview over the telephone in a quiet environment. The questionnaire and interview outlines are listed in Additional File 1. Three researchers including two master candidates (QJ and ML) and an MD candidate (YS) with qualitative research skills and established relationships with the patients in the previous registry study conducted the interviews. All participants were informed of the purpose of the study before data collection began. The interviews were audio-recorded with the consent of the interviewees, and the interviewers took field notes during the study period. Interviewers were required to repeat the answers to confirm the participants’ expressions, and transcripts were not returned to the participants. All interviews were single. Once the patient felt severe fatigue, difficulty in breathing or talking, or otherwise could not finish the interview, it was postponed. Data collection was discontinued when all the researchers achieved consensus on data saturation.

### Data analysis

Data from CARE-TCM and the questionnaires were described statistically. Audio recordings were transferred into text and stored anonymously. Interview transcripts were analysed thematically, allowing the combination of inductive and deductive analyses simultaneously [18]. We used qualitative analysis software Nvivo11.0 to aid in the analysis process. Data analysis began with the first interview, and the emerging results shaped subsequent sampling decisions. Transcripts were coded based on the interview guide. For consistency, two researchers (QJ and YS) analysed the first three cases. All researchers (QJ, YS, and ML) were required to be familiar with the entire interview through audio recordings, transcripts, and field notes. The labels coded by two of the researchers (QJ and YS) were compared and used to develop a set of codes with the consensus of all three researchers (QJ, YS, and ML) to apply to subsequent analysis. The codes were grouped into categories, generating a primary framework containing prior and emergent concepts. The framework was enriched in the next analytic process until no more themes arose from the interviews. Reflexivity, rigour, and quality are requisite issues in this study, as in other qualitative methods.

### Rigour

The study report follows the Mixed Methods Appraisal Tool (MMAT) version 2018 [19]. The checklist is listed in Additional File 2.

## Results

### Questionnaire

Phase I included a total of 52 patients with ALS using TCM who had been followed up for over six months. The baseline clinical parameters gathered from CARE-TCM are summarised in Table 1. Participants’ ages ranged from 30 to 83 years. Chinese herbs were the most commonly chosen modality by all patients, with 88.5% of them also taking riluzole alongside TCM treatment. The treatment modalities used by patients are listed in Table 2. The questionnaire showed that the majority of patients chose to continue TCM treatment. Eight patients stopped TCM, with six of them making this decision after three months of receiving TCM.

**Table 1:** Demographic data and clinical information gathered from CARE-TCM^a^

	Number (Percentage)
Total number of participants	52 (100.0%)
Age	
<40 years old	4 (7.7%)
40–59 years old	30 (57.7%)
≥ 60 years old	18 (34.6%)
Gender	
Female	17 (32.7%)
Male	35 (67.3%)
ALSFRS-R^b^	
40–48	27 (51.9%)
30–39	15 (28.8%)
20–39	7 (13.5%)
<20	3 (5.8%)

^a^ CARE-TCM: China Amyotrophic Lateral Sclerosis Registry of Patients with Traditional Chinese Medicine. ^b^ ALSFRS-R: total score 48. ^c^ TCM: traditional Chinese medicine

**Table 2:** Modalities of treatment patients use

	Baseline	Current usage
Total number of participants	52 (100.0%)	
TCM^a^	52 (100.0%)	44 (84.6%)
Chinese herb	52 (100.0%)	44 (84.6%)
Moxibustion	8 (15.4%)	8 (15.4%)
Acupuncture	18 (34.6%)	1 (1.92%)
Massage	10 (19.2%)	10 (19.2%)
Chinese exercise	1 (1.9%)	0 (0.0%)
Riluzole	46 (88.5%)	45 (86.5%)
Edaravone	21 (40.4%)	0 (0.0%)^b^

^a^ TCM: traditional Chinese medicine. ^b^ Edaravone is administrated for 6 months, while filling the questionnaire the 21 patients have finished theirtreatment

### Semi-structured interview

In Phase II, 16 patients received a telephone interview until data saturation was reached. All patients we intended to interview participated in this study. The interview sessions averaged 35 min (range: 19–69 min). The demographics of the participants are listed in Table 3. The interviews yielded two main themes and eight subthemes, which are listed in Table 4.

**Table 3:** The demographics of patients and interviewees

Patient	Gender	Age	Time of oneset	Time of receiving TCM^a^	Continue TCM	Riluzole	Participant	ALSFRS-*R*^b^
I	female	53	2013.02	2014.07.01	Yes	Yes	caregiver	29
II	male	46	2021.02	2022.08.31	Yes	No	patient	42
III	male	45	2018.06	2019.12.04	Yes	No	caregiver	22
IV	male	56	2018.12	2019.07.17	Yes	Yes	patient	12
V	male	64	2014.08	2019.01.09	Yes	Yes	caregiver	16
VI	female	46	2019.10	2022.09.28	Yes	Yes	patient	42
VII	female	49	2021.01	2021.12.08	Yes	No	patient	None^c^
VIII	female	39	2016.01	2022.08.03	Yes	No	patient	42
IX	male	65	2016.04	2018.08.29	Yes	Yes	caregiver	20
X	male	35	2021.05	2022.08.10	No	Yes	caregiver	35
XI	female	57	2021.03	2022.07.13	Yes	Yes	caregiver	40
XII	male	46	2021.03	2022.07.13	No	Yes	patient	40
XIII	female	75	2020.01	2021.05.31	Yes	Yes	caregiver	8
XIV	male	30	2020.10	2021.05.26	No	Yes	caregiver	2
XV	male	47	2019.12	2021.03.01	No	Yes	caregiver	8
XVI	male	51	2013.03	2021.04.16	Yes	Yes	caregiver	24

^a^ TCM: traditional Chinese medicine. ^b^ ALSFRS-R: Amyotrophic lateral sclerosis functional rating scale-revised. ^c^ The patient was too anxious to fill the questionnaire

**Table 4:** Themes and subthemes raised from the interview

Themes	Subthemes
Reasons for using TCM^a^	1) Limited treatment options 2) Traditional concepts 3) Holistic regulation 4) Experience from wardmate 5) Economic factors
Experience of TCM	1) HM^b^ is preferred 2) Recognising the ineffectiveness of TCM 3) Challenges for TCM

^a^ TCM: traditional Chinese medicine. ^b^ HM: herbal medicine

#### Reasons for using TCM

Patients’ reasons for using TCM were grouped as five subthemes encompassing limited treatment options, traditional concepts, holistic regulation, experience from wardmate and economic factors.

##### Limited treatment options

ALS remains a fatal disease. Although there are FDA-approved medications intended to delay disease progression and extend lifespan, they do not provide a satisfactory curative effect. Thus, patients desire to try any treatments that are available.

*I also heard what others said and read it online, but I don’t know if TCM has any effect. To put it bluntly, as the old saying goes, a drowning man will clutch at a straw. Just have a try. (Participant IX, caregiver)*

Participant X also expressed a sense of powerlessness and a willing to cure disease.

*We just want to have a try. Because there are no good ways in conventional medicine now, we are all groping. We don’t know which is better, but we hope to delay disease progression and hope to cure it. If there was a better way now, we’d try it, regardless of how expensive it was. (Participant X, caregiver)*

Participant XII tried herbs despite having never used TCM before and had no expectations towards TCM.

*In the beginning, I didn’t have any expectations for TCM. You know, I just tried it. (Participant XII, patient)*

Concern for side effects and efficacy of conventional medicine has led to some patients taking only HM. Participant III refused conventional treatment as she stated TCM has fewer side effects than conventional medicine.

*If I were to take a long-term medication, I would choose Chinese herbs. At least it doesn’t have so many side effects. I agree with Chinese herbs combined with conventional medicine. If conventional medicine is absolutely beneficial and harmless for this disease, then I will definitely take it. You see, there are so many side effects on the package insert. I won’t take this kind of drugs. (Participant III, caregiver)*

##### Traditional concepts

TCM has been practiced in China for over two thousand years. Patients place their hope in it owing to cultural beliefs and ingrained thinking. Some patient resort to TCM because they have a habit of using it in their daily lives to treat illnesses.

*Well, we usually treat illnesses using TCM. Since there are only a few conventional medicines, we have no other choice and have to take herbs. (Participant XI, caregiver)*

Combining riluzole with TCM is common among patients with ALS. Participant V holds the assumption that conventional medicine works more quickly, whereas Chinese medicine works more slowly and cures the primary symptom.

*It is said Western medicine works more quickly, while Chinese medicine works more slowly and cures the root cause. Maybe combining the two treatments would be better. (Participant V, caregiver)*

Some patients did not even regard “soaking herbs in water” as a form of TCM treatment. Participant IX said that the patient was given water with *Astragalus propinquus* Schischkin, known as *Huangqi*, in TCM every day. Experimental studies on animal models have verified the neuroprotective effects of *Huangqi* in neurodegenerative diseases [12].

*No, he didn’t receive TCM…He drank water with Huangqi every day these years. I just soak it in the water. I found it appeared in all the prescriptions we received from the hospital. (Participant IX, caregiver)*

##### Holistic regulation

Recent studies have confirmed that patients with ALS experience both motor and nonmotor symptoms, including sleep disturbance, mood disorders, excessive sweating, and pain [20]. However, treatment options for nonmotor symptoms in ALS are still lacking. Many participants believe that TCM’s holistic approach helps regulate patients’ overall wellbeing. While using HM, they felt physically and mentally comfortable, although motor symptoms continued to progress.

*Well, I think it should be effective. Anyway, my whole body felt quite comfortable when I took Chinese herbs. Although there was no obvious improvement (in motor function), I want to stick to it for a while. (Participant VI, patient)*

Participant I emphasised the role of TCM in patients’ quality of life (QOL).

*The improvement was holistic rather than in a particular symptom. She is more energetic and not as tired as before. The QOL is definitely better after taking herbs. In general, there were improvements, both physically and mentally, but not in a particular symptom. (Participant I, caregiver)*

Patients are concerned about the treatment of non-motor symptoms. Participant II found that TCM improved appetite and relieved constipation. Loss of appetite is linked to undesirable weight loss [21], which has been identified as a negative prognostic factor influencing mortality and morbidity in patients with ALS [22].

*As for my appetite, I used to have heartburn sometimes, but now I have none. My stool is now normal, and the bitter taste in my mouth also improved a little. (Participant II, patient)*

Participant VIII reported an improvement in hyperhidrosis.

*I used to sweat all the time. After taking herbs, I no longer sweat as much as before. Anyway, I’m still going to take herbs.(Participant VIII, patient)*.

##### Experience from wardmate

Patients’ perceptions and experience, especially those shared by their wardmates, influence their attitude towards TCM. Participant II maintained TCM because of the experiences of other patients.

*At first, I didn’t know much about this disease. I was very scared at that time. I joined a group in which patients suffer from the same disease, and most of them said that riluzole had no effect. Later on, I saw a message that a patient’s condition was alleviated by taking Chinese medicine, so I stuck with it. (Participant II, patient)*

Participant VII opted exclusively for Chinese herbs due to the unsatisfactory efficacy of conventional remedies for other patients.

*A friend told me he took riluzole, but the efficacy is unsatisfying. So, I tried Chinese herbs. I would like to use Chinese herbs only. The medicine (riluzole) is useless. So many celebrities would be cured if it works. (Participant VII, patient)*

Participant XII, who is a doctor initiates HM because of family members’ and fellow workers’ influence, despite holding a resistant attitude towards TCM.

*I had no expectations (to TCM). I thought it was of little use. My wife and colleagues persuaded me to have a try in case it helps. (Participant XII, patient)*

##### Economic factors

Patients with ALS always use medicines from the point of diagnosis to their death, posing an economic burden for their families.

*I don’t know if we should continue the treatment. Taking riluzole does not improve the disease. We are in a pinch financially. Medicines are too expensive. (Participant XIV, caregiver)*

The financial condition affects patients’ decisions on treatment. Medications are expensive, and medical insurance policies vary throughout the country. Patients who distrust both TCM and conventional medicine tend to opt for more affordable treatment. For Participant IX, riluzole is cheaper.

*Riluzole is enrolled in the medical insurance medicine list in my city. We only need to pay 10% of the price. Chinese herbs are quite expensive and not reimbursable. (Participant IX, caregiver)*

For Participant VIII, the price of HM is more affordable.

*I only take herbs. The price of combining Chinese herbs and riluzole is too high for me. Riluzole is too expensive. If it is cheaper, I would try it. (Participant VIII, patient)*

#### Experience of TCM

We explored patients’ experiences with TCM and acquired three subthemes, including HM is preferred, recognising the ineffectiveness of TCM and challenges for TCM.

##### HM is preferred

TCM comprises various modalities, with HM being the most commonly chosen option in our patient group. However, the quality of herbs is a concern, and decocting herbs is cumbersome. Participant II felt the quality of herbs varies among hospitals.

*The herbs from a nearby hospital were not as good as the herbs from your hospital, in taste and other aspects. (Participant II, patient)*

Participants pay close attention to maximise the efficacy of HM. Though Chinese herb granules and hospital decoctions are available and are more convenient, decocting by themselves is often preferred.

*It’s definitely inconvenient to decoct it by myself, but I will be more reassured. Granules are convenient, but the quality is not trustworthy for me. (Participant III, caregiver)*

Massage, moxibustion, and cupping were mentioned in some cases but were not suitable for everyone. Muscle atrophy caused by disease progression often makes massage intolerable.

*My father is particularly thin and has not exercised for a long time. Even if the masseur uses a slightly heavier massage technique, he would still feel pain. (Participant V, caregiver)*

*It was true that there was a positive trend in the first month of massage. It is achievable. When he felt muscle soreness, with the aid of massage and moxibustion, this problem was solved. (Participant III, caregiver)*

Chinese exercises such as Taiji and Baduanjin are difficult for patients with disabilities as they require using their entire bodies. Participant VI used to do Baduanjin, but now she is afraid of falling and has refused to practice the exercises independently.

*I’m afraid I can’t stand firm. In the beginning, I practiced Baduanjin, but I dare not practice it independently now. I’m afraid I’ll fall. (Participant VI, patient)*

Most of the patients who have ever used acupuncture (*n* = 18) discontinued the treatment(*n* = 17). One reason might be that acupuncture could only be conducted by trained persons. Another reason could be that the effect of acupuncture is controversial. One participant in this study who tried acupuncture felt the weakness worsened.

*I received acupuncture for about two weeks when I first got sick. However, after two weeks of acupuncture, my leg got noticeably worse, and the progression of the disease accelerated. (Participant IV, patient)*

##### Recognising the ineffectiveness of TCM

Currently, no effective treatment has been found that could stop the disease process. Many patients who have recognised the ineffectiveness of TCM in curing ALS discontinued HM.

*I took herbs intermittently for about three months. After that, I only took riluzole and didn’t take herbs anymore. There is no evidence to prove its efficacy. I’m resistant to TCM. (Participant XII, patient)*

According to the result of the questionnaire, eight patients stopped TCM treatment. Notably, six of them made this decision after a three-month trial, indicating the first three months of treatment might be an important period.

*There was no obvious effect after taking Chinese medicine for three months. Whether there is an effect determines everything. When there is no effect, continuing to take traditional Chinese medicine is meaningless. (Participant XI, caregiver)*

##### Challenges for TCM

Combining the four diagnostic methods of inspection, auscultation and olfaction, inquiry, and palpation, provid-ing treatment on the basis of pattern identification is an important part of TCM. However, disability in patients with ALS makes it difficult for them to visit the doctor. They often contact the doctor over the phone via video calls, making palpation impossible.

*He can’t move anymore and could only receive remote medical treatment. It’s too inconvenient for him to go to Beijing. To see the doctor via a video call over the phone is more suitable for him. (Participant V, caregiver)*

*I think going to Beijing for a face-to-face diagnosis would be better than video calls because TCM practitioners cannot accurately observe tongue coating through video and cannot feel the pulse. (Participant XI, caregiver)*

The traditional mode was preferred by the participants. Even if they are unable to travel, participants value seeing the doctor face-to-face.

*If permitted, we prefer to go to the hospital. I think an on-site visit helps to better understand the patient’s condition. (Participant I, caregiver)*

This inconvenience has even led to HM no longer being used by some patients.

*To go to Beijing is inconvenient. This is the main problem for us. We have to look after our children. His legs are paralysed. TCM practitioners need to look, listen, question, and feel the pulse. I think this is limited if the doctor can’t see the patient face-to-face. (Participant X, caregiver)*

Furthermore, Participant XIV expressed a willingness to receive HM, but they do not have access to a doctor. Due to the severe condition of the patient, he could not live without a non-invasive ventilator, making it challenging to travel long distances. Yet, the uneven distribution of medical supplies makes it challenging to obtain HM.

*As long as you can see a doctor, it’s great. Whether through video call or at the hospital. However, we have no doctor. What this means is that we can’t seek medical treatment for this disease in our county. (Participant XIV, caregiver)*

## Discussion

This study has identified that patients with ALS resort to Chinese herbs to holistically regulate their bodies owing to limited treatment options. Notably, a common reason mentioned by all the participants who adhere to TCM is the feeling of comfort, even though muscle atrophy is not effectively improved. This finding suggests that nonmotor symptoms and QOL are substantial concerns for patients with ALS, in addition to lifespan. Studies have shown that patients with ALS often experience nonmotor symptoms such as neuropsychiatric, autonomic, vascular, and gastrointestinal disturbances, which are unrelated to underlying motor pathology [20]. A growing body of evidence suggests that the burden of nonmotor symptoms is a major determinant of QOL [23]. QOL represents a self-perceived outcome that reflects the patient’s perspective [24]. In addition, a cross-sectional study involving 58 patients found a positive correlation between nonmotor symptoms and disease progression [25]. Although the relationship between QOL and survival in patients with ALS remains unclear, higher QOL has been associated with improved survival in other diseases with terminal prognoses [26].

Moreover, we found that among TCM treatment modalities, HM was the commonest way that might be a more suitable option for patients with ALS. Traditional Chinese exercise, which requires whole-body coordination, may not be suitable for patients with disabilities. However, a systematic review found that therapeutic physical exercise could slow down muscle deterioration in patients with ALS, as measured by ALSFRS-R and functional capacities [27]. This suggests that Chinese exercises could be a viable treatment option with necessary modifications to accommodate patients with limited mobility. Massage was reported as a potential strategy for managing stiffness, muscle soreness, or muscle pain, but it was mainly carried out by patients’ family members rather than trained technicians. Clinical studies on massage in ALS are rare, and massage is usually integrated with herbs or acupuncture. Therefore, further investigation is needed to determine the efficacy, components, manual manipulation, frequency, and duration of massage. As for acupuncture, the results of the questionnaire showed that only one patient continued this treatment, and notably, he felt that disease progression was accelerated. A scoping review of acupuncture in ALS found that most studies reported significant efficacy, with only a few studies reporting adverse events [28]. However, the inclusion of case reports and case series in this review, rather than higher-quality study designs, may reduce the reliability of the result. The use of acupuncture in ALS remains controversial.

The experience of the first three months of receiving HM might be important, as it can have a substantial impact on patients’ decision-making. Although previous randomised controlled trials usually set the intervention period at six months, it is worth noting that most participants who discontinued HM in our patient group made their decision at the three-month mark. This finding suggests that the first three months may be an important period in patients’ psychological expectations, which should be considered in further clinical studies.

The use of HM among patients with ALS faces challenges as the disease progresses. In TCM, combining the four diagnostic methods and prescribing individualised based on pattern identification are important processes, as emphasised by patients. However, limited physical mobility due to the disease often makes it difficult for patients to visit doctors, resulting in some patients discontinuing HM. ALS is a rare disease with affected patients scattered across regions, which makes the imbalance of medical resources between regions more prominent. Despite the implementation of a classified diagnosis and treatment system in China in 2015, its effectiveness remains unproven [29]. The medical system should promote hierarchical diagnosis and treatment to make it more accessible for patients with disabilities. Moreover, artificial intelligence-based TCM diagnosis has rapidly grown in recent years, potentially enabling remote diagnosis, prescription, and standardisation for TCM [30].

Given the lack of effective treatment options, economic condition can have a considerable impact on patients’ treatment decisions. Disease-related work absences lead to income loss, while medication expenses, equipment, mobility aids, and caregiver services strain the financial resources of patients and their families. Basic insurance policies vary throughout China, and financial assistance methods should be explored. A previous study concluded that out-of-pocket expenses incurred by patients are substantial, illustrating the need for additional resources to support affected families [31].

HM is widely used for ALS, not only in China. A survey showed that 42% of ALS patients in the United States [32], and 46.7% in Korea use HM [33]. A systematic review and meta-analysis of HM for ALS, which included twenty studies, suggests that HM seems to produce superior treatment responses for ALS without an increased risk of adverse events. However, the quality of evidence was generally low [34]. Furthermore, a comparison between HM users and non-HM users based on propensity score matching using the largest ALS clinical trials dataset (the Pooled Resource Open-Access ALS Clinical Trials Database, PRO-ACT) indicated that the use of HM prolonged overall survival within 18 months and improved activity limitation [35].

However, the quality of HM is of great concern, not only for patients with ALS. Multiple factors, including contamination, adulteration, misidentification, growing conditions, and geographical region, affect the concentration of active ingredients in plant materials and products. Most participants prefer decocting herbs themselves rather than consuming Chinese herb granules or herbs decocted by a hospital. Herb granules are extracted from herbs separately and lack the interactive process that decocting provides. Although a meta-analysis comparing the efficacy of herb granules and traditional decoction concluded that herb granules performed better, the low quality of the included studies and potential publication bias cast doubt on this conclusion [36]. Ensuring the quality and stability of HMs presents severe challenges for the herbal industry [37]. The selection and processing of raw materials, production technology, quality control, and consistency evaluation of TCM formula granules need improvement [38]. The equivalence of decoction and formula granules also requires further verification [39].

Notably, a particular herb, Huangqi, was mentioned by a participant as a consistent ingredient in every prescription. From a TCM perspective, ALS is characterised by voluntary muscle weakness, atrophy, and flaccidity, with spleen deficiency playing an essential role in disease progression [40]. With spleen deficiency, the function of nourishing the muscle is affected, leading to muscle atrophy and flaccidity. Huangqi is widely used in Chinese decoction to tonify the spleen and nourish Qi. Pharmacological studies have sub-stantiated that Huangqi can combat neurodegeneration by inhibiting apoptosis, synaptotoxicity, and mitochondrial dysfunction [41, 42], which are considered potential pathological mechanisms in ALS [43].

### Limitation

This is the first mixed method study on the reasons and experiences of patients with ALS using TCM. Despite being conducted based on the largest ALS registration study of patients with TCM, the sample size was limited as ALS is a rare disease with a low incidence and prevalence. Progression of the disease resulted in dysarthria. Consequently, ten patients were primarily interviewed through their caregivers. However, the caregiver’s responses may not fully represent those of the patients. To minimise the impact of caregivers’ viewpoints on the study, patients were required to be present during the interview, and no significant differences were found between patients and caregivers during the analysis of interview data. A further limitation is that the study only recruited Chinese patients, thus excluding the views of patients from other countries. However, the study was performed in China, where TCM originated, and the proportion of TCM users among patients with ALS is significantly higher than that in other countries. Thus, Chinese patients have greater access to a variety of TCM treatments and may possess a more holistic understanding of TCM.

## Conclusion

Patients with ALS resort to TCM for holistic regulation owing to limited pharmaceutical options and cost. Our findings highlight the significance of TCM in treating nonmotor symptoms and improving QOL. Most patients choose HM as their preferred TCM treatment modality, considering it more suitable. However, there is a need to enhance the quality control of HM. Disease progression leads to disability, posing challenges to patients seeking HM therapy. It is important to promote the implementation of a hierarchical diagnosis and treatment approach to provide a more convenient way for patients to access TCM treatment. In summary, TCM based on pattern identification holds significance in ALS treatment, which is also reinforced by patients as a reason for using TCM. While, there still need future clinical studies to explore the potential of HM in improving nonmotor symptoms and enhancing QOL.

### Electronic supplementary material

Below is the link to the electronic supplementary material.

Supplementary Material 1

Supplementary Material 2

## Data Availability

The datasets supporting the conclusions of this article are included within the article and its additional files.

## Abbreviations

ALS: Amyotrophic lateral sclerosis
FDA: Food and drug administration
TCM: Traditional Chinese medicine
CARE-TCM: China Amyotrophic Lateral Sclerosis Registry of Patients with Traditional Chinese Medicine
HM: Herbal medicine
ALSFRS-R: Amyotrophic lateral sclerosis functional rating scale-revised
QOL: Quality of life
PRO-ACT: The Pooled Resource Open-Access ALS Clinical Trials Database
