# Supplementary material for: Reasons and experience for patients with amyotrophic lateral sclerosis using traditional Chinese medicine: a CARE-TCM based mixed method study
Source: BMC Complement Med Ther. 2024 Jun 12;24:231. doi: 10.1186/s12906-024-04513-2 (PMC11167840; doi:10.1186/s12906-024-04513-2)
Supplement: Supplementary file 1 — Supplementary Material 1 [file 12906_2024_4513_MOESM1_ESM.docx]

**Questionnaire**

| **What type of therapies are you using to treat ALS?**  *Have you ever used any therapies listed below and have discontinued them? Please fill in the blank with the duration of your usage. | | |
| --- | --- | --- |
| TCM^a^ | | How long did you use it? |
| YES | NO |  |
| Chinese herb |  |  |
| YES | NO |  |
| Moxibustion |  |  |
| YES | NO |  |
| Acupuncture |  |  |
| YES | NO |  |
| Massage |  |  |
| YES | NO |  |
| Chinese exercise |  |  |
| YES | NO |  |
| Riluzole |  |  |
| YES | NO |  |
| Edaravone |  |  |
| YES | NO |  |

^a^ TCM: traditional Chinese medicine.

**Interview outline**

| Interview outline |
| --- |
| 1. Have you ever treated with conventional medicine? How did you feel? 2. How did you get to know TCM? What motivated you to seek TCM treatment? 3. What were your expectations regarding TCM? 4. Which form of TCM have you ever tried? 5. How did you feel after receiving TCM treatment? 6. Did you think TCM act a role? What did TCM display? 7. Which treatment do you prefer in the future? Why? 8. Have you ever met any difficulties while taking TCM? 9. What factors do you think affecting the symptoms while taking TCM? 10. Do you have any other comments or advices about TCM? |
